# Periapical implant lesion: A systematic review

**DOI:** 10.4317/medoral.21698

**Published:** 2017-10-21

**Authors:** Juan-Antonio Blaya-Tárraga, Juan Cervera-Ballester, David Peñarrocha-Oltra, Miguel Peñarrocha-Diago

**Affiliations:** 1 DDS, MSc. Master of Oral Surgery and Implantology. University of Valencia Medical and Dental School. Valencia, Spain; 2 DDS, MSc. Collaborator Professor Oral Surgery. University of Valencia Medical and Dental School, Spain; 3 DDS, MSc, PhD. Assistant Professor Oral Surgery. University of Valencia Medical and Dental School, Spain; 4 MD, MDM, PhD. Chairman of Oral Surgery, Director of Master in Oral Surgery and Implantology. University of Valencia Medical and Dental School, Spain

## Abstract

**Background:**

The aim of this study was to systematically review the evidence for periapical implant lesion, which makes a patient more susceptible to the periapical lesion, frequency, symptoms, signs (including radiological findings) and possible treatment options.

**Material and Methods:**

A systematic literature review and analysis of publications included in PubMed, Embase and Cochrane; articles published until March 2016; with a populations, exposures and outcomes (PEO) search strategy was performed, focused on the issue: “In patients with periapical lesion to the implant during the osseointegration, what symptoms, signs, and changes in complementary examination manifested, for according to that stage, be intervened with the appropriate approach?”. The set criteria for inclusion were peer-reviewed articles.

**Results:**

From a total of 212 papers identified, 36 studies were included in this systematic review, with 15461 implants evaluated and 183 periapical implant lesions. Which 8 papers included more than 5 cases and 28 included equal or less than 5 cases. Analysis of the papers revealed that periapical implant lesion is classified according to evolution stages into acute (non-suppurated and suppurated) and subacute (or suppurated-fistulized). In the acute stage and in the subacute if there is no loss of implant stability, the correct treatment approach is implant periapical surgery. In the subacute stage associated with implant mobility the implant must be removed.

**Conclusions:**

Evidence on the subject is very limited, there are few studies with small sample, without homogeneity of criteria for diagnosing the disease and without design of scientific evidence. Currently etiology lacks consensus. The early diagnosis of periapical implant periapical lesions during the osseointegration phase and early treatment, will lead to a higher survival rate of implants treated, hence preventing the need for implant extraction.

** Key words:**Apical peri-implantitis, retrograde peri-implantitis, inflammatory peri-implantitis lesion.

## Introduction

Periapical implant lesion, also referred to as apical peri-implantitis or retrograde peri-implantitis, was first described by McAllister in 1992 (1) as an injury that occurs in the apical portion of an implant, causing failure of osseointegration. Sussman & Moss (2) defined it as the infectious-inflammatory process of the tissues surrounding the implant apex; and Quirynen *et al.* (3) as a clinically symptomatic periapical lesion that develops shortly after implant insertion while the coronal portion of the implant achieves a normal bone to implant interface.

The etiology of this lesion is not yet clear; however, several factors have been proposed that could be related to the onset of pathology. For some authors the most likely cause is the endodontic pathology of the tooth replaced by the implant or of the adjacent tooth (4-8). Other factors described were contamination of the implant surface (9,10), bone overheating during milling or preparation greater than the necessary for the implant (9,11,12) and pre-existing bone disease, presence of root fragments or foreign bodies (5,9,12).

The aim of this systematic review was to assess the papers to describe the concept, frequency, etiology, diagnosis, clinical classification, surgical procedure and prognosis.

## Material and Methods

This systematic review was conducted in accordance with Preferred Reporting Items for Systematic Reviews and Meta-Analisis (PRISMA) (13). The study design was determinated with a protocol by the authors before the review process.

- Focused question

Search strategy was performed with populations, exposures and outcomes (PEO) to synthesize the next focused question: “In patients with periapical lesion to the implant during the osseointegration, what symptoms, signs, and changes in complementary examination develop, for according to that stage, be intervened with the appropriate approach?” ( Table 1).

**Table 1:**
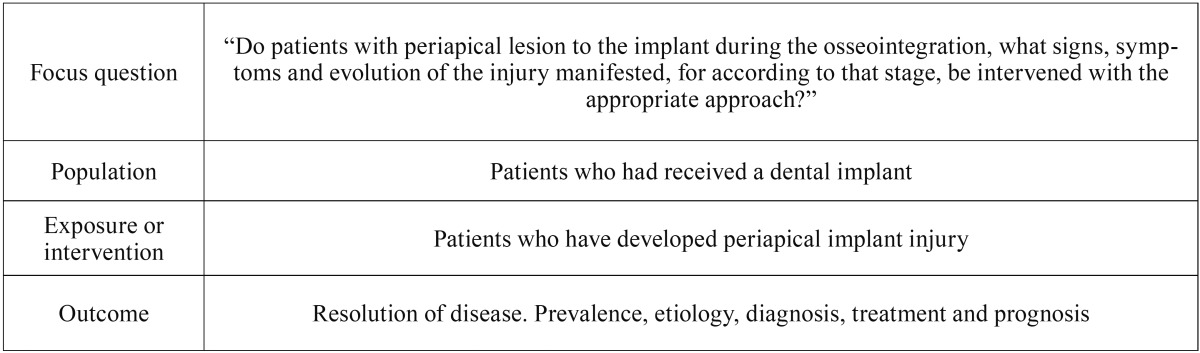
Population, exposure, and outcomes (PEO) strategy.

- Search method for identification of studies

Scientific papers were searched in PubMed, Web of Science and Cochrane. Manuscripts published until March 2016 were included. The following query was used: ((((apical peri-implantitis)) OR (retrograde peri-implantitis)) OR (inflammatory peri-implantitis lesion)).

- Inclusion and exclusion criteria

Inclusion criteria were as follow: (1) populations: all human controlled clinical trials and prospective or retrospective clinical studies; (2) exposures: patients who have been studied the development of early failure or develop at least one periapical lesion implant, describing pathological condition and clinical intervention; (3) outcomes: frecuency of the lesion, etiology, diagnosis, treatment and prognosis.

Exclusion criteria were as follow: (1) articles describing coronal peri-implantitis; (2) delayed complications or late implant failure; (3) reviews or update reviews.

- Data extraction and analysis

Titles and abstracts from the three search engines were downloaded to Mendeley software (Elsevier Inc, NY, USA). Mendeley was used to import the reference data, and to manage the imported references. Two reviewers (JB, JC) screened titles and abstracts independently of each other. Disagreement regarding inclusion was resolved by discussion. Full text manuscripts of the selected studies were obtained and further reviewed for inclusion. These were inserted into an excel work sheet. Papers were divided into two groups, with more than 5 cases and less than 5 cases.

Most of the included studies are observational studies without data collection protocols, case report or case series, with high risk of bias.

## Results

- Search results and study description

**Figure 1:**
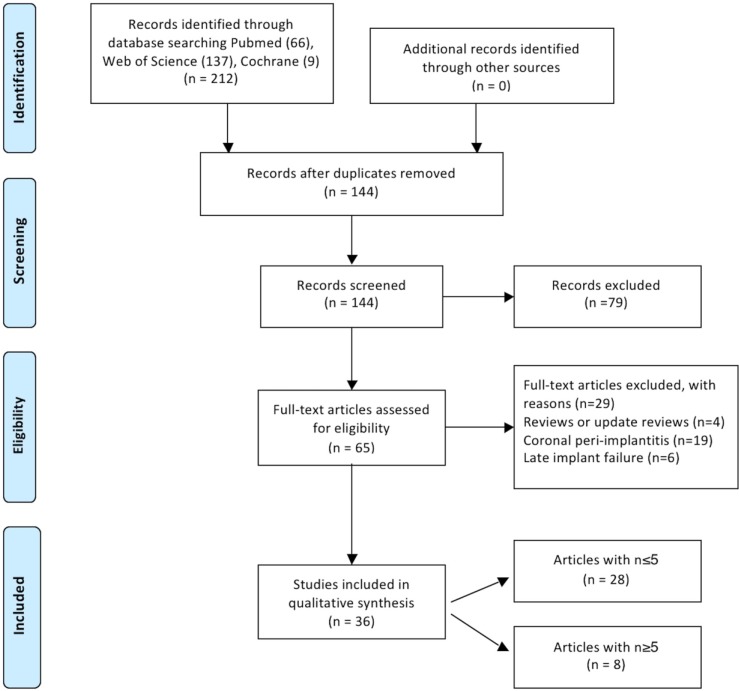
Flow chart diagram of screened withdrawn and included articles through the review process.

A total of 212 abstracts were retrieved and evaluated independently by two reviewers. A total of 36 publications were selected for the purpose of the systematic review (Fig. 1). Were evaluated a total of 15.461 implants, of which 183 periapical implant lesions were described. 8 publications included more than 5 cases ( Table 2, Table 3) and 28 included 5 cases or less ( Table 4, Table 4 continue, Table 5,  Table 5 continue). The studies published were conducted at universities (30/36), hospital (3/36) or private practice centers (3/36).

**Table 2:**
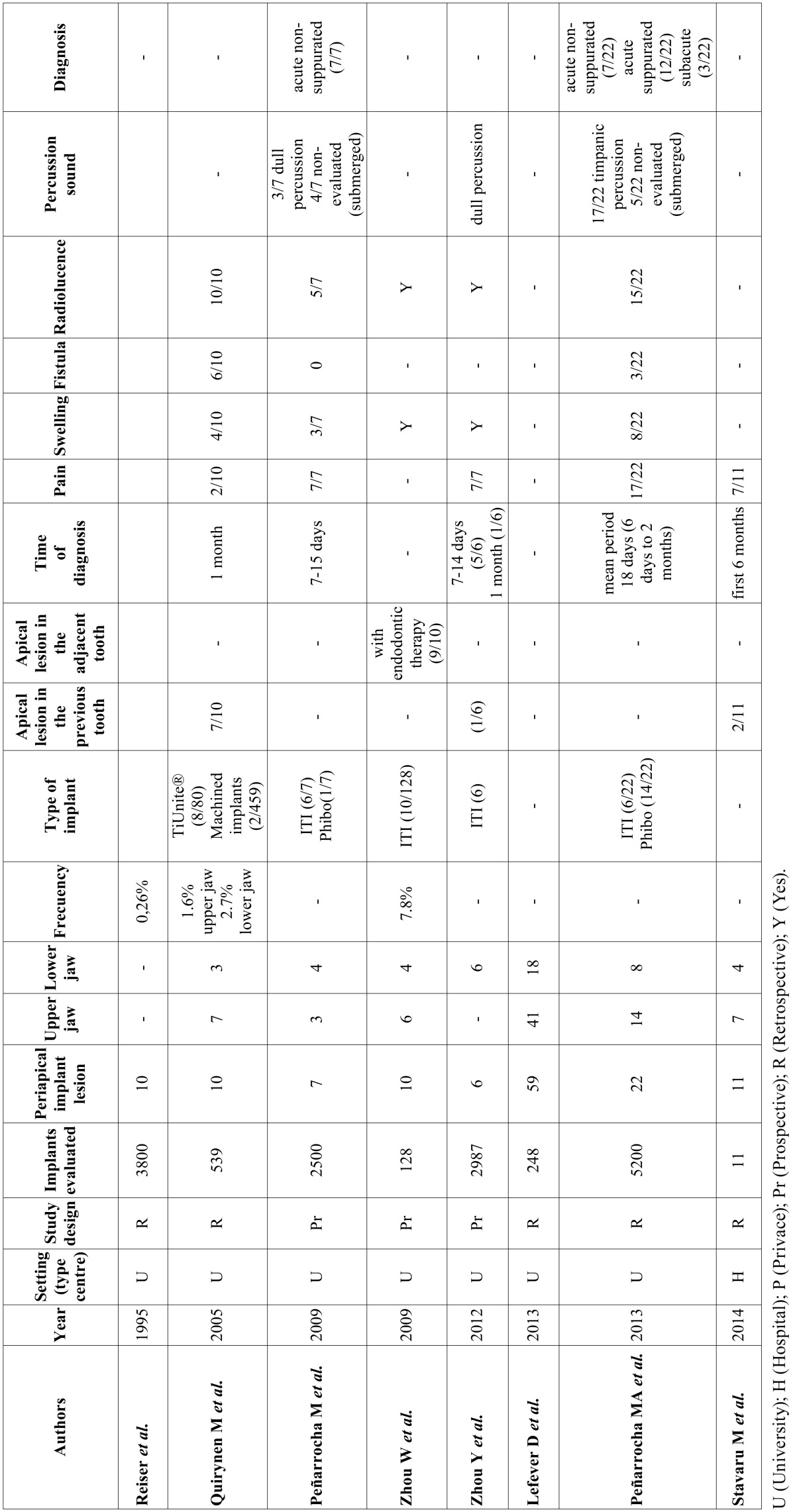
Articles with n≥5, study design and diagnosis.

**Table 3:**
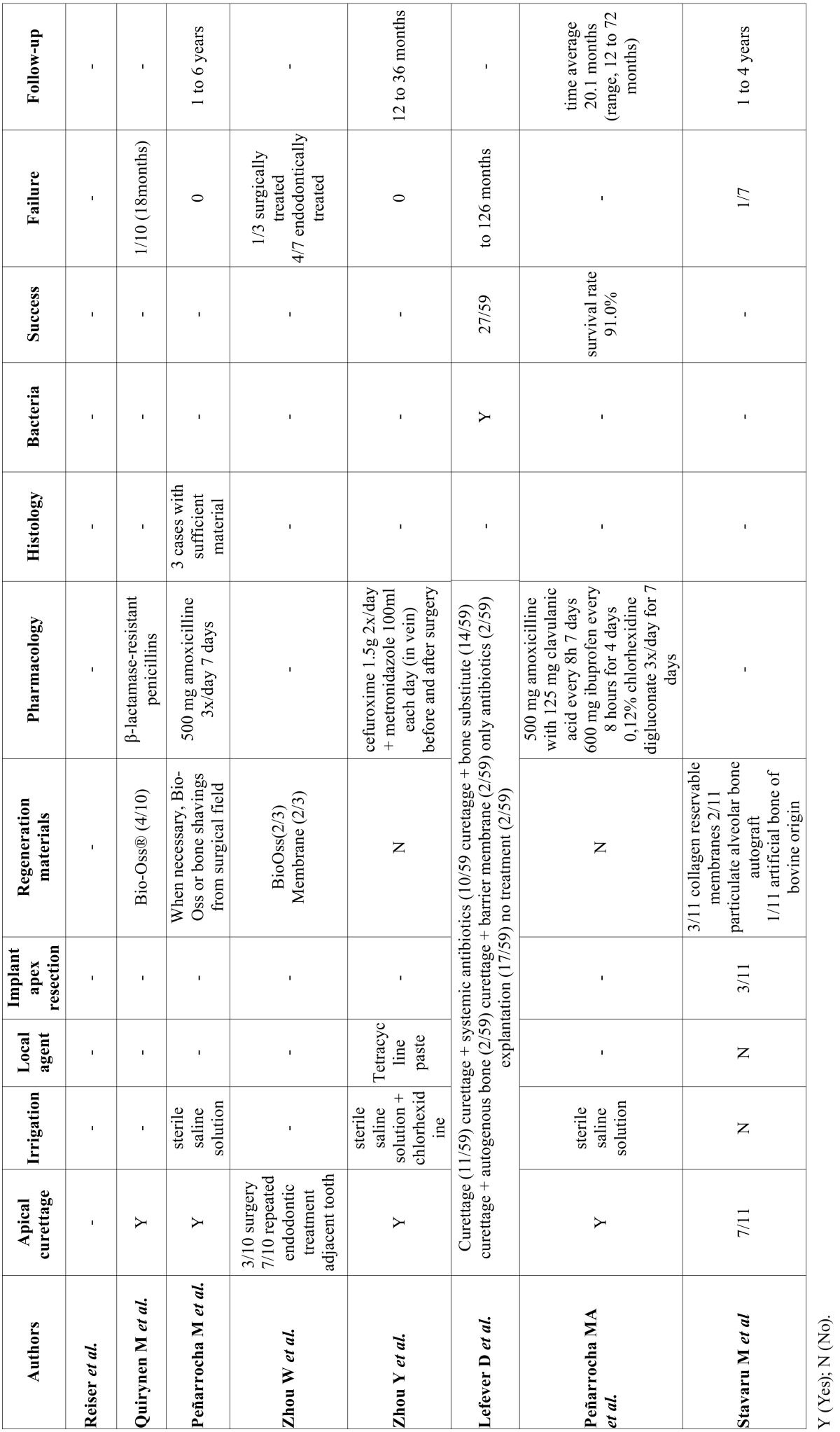
Articles with n≥5, treatment and follow-up.

**Table 4:**
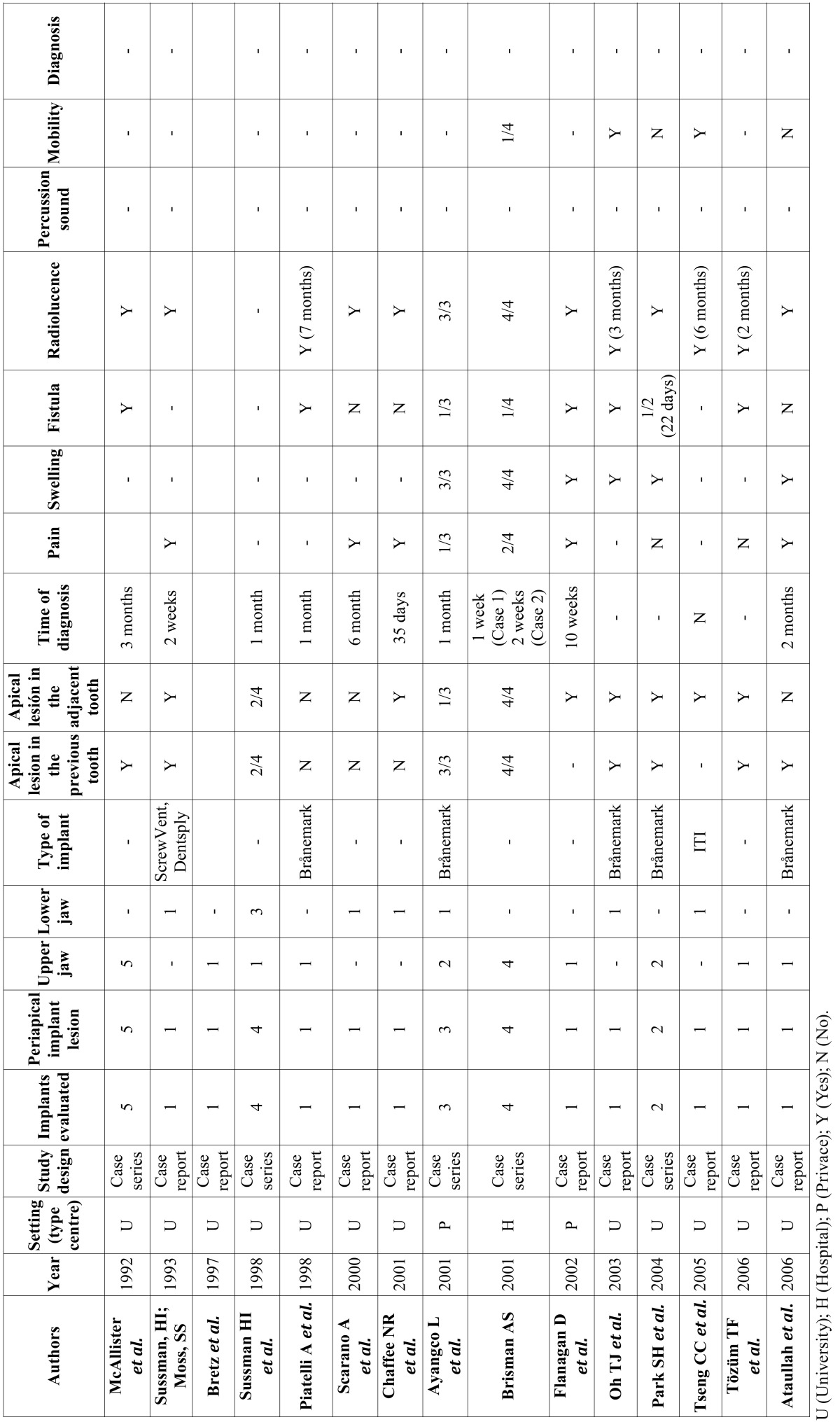
Articles with n<5, study design and diagnosis.

**Table 4 continue:**
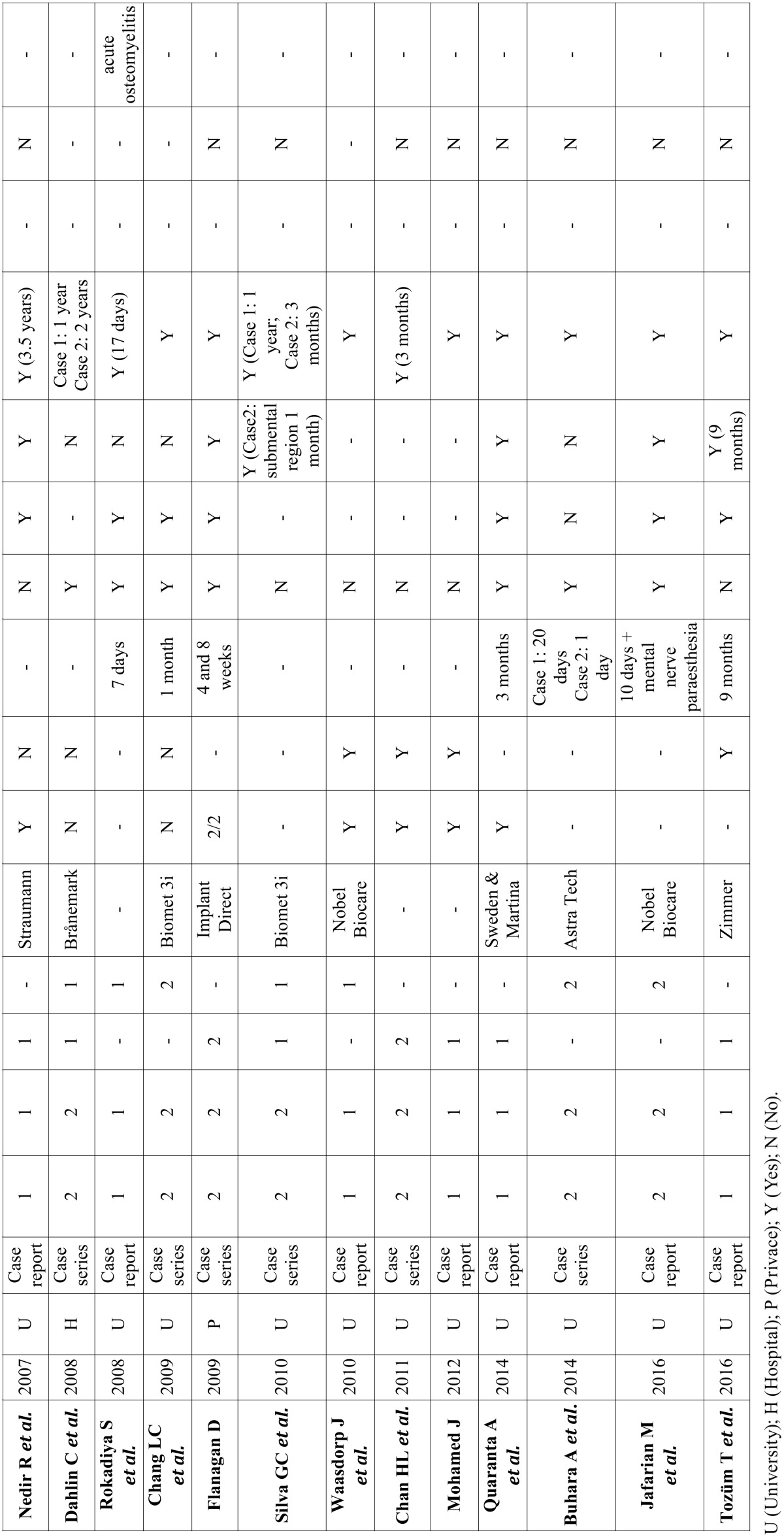
Articles with n<5, study design and diagnosis.

**Table 5:**
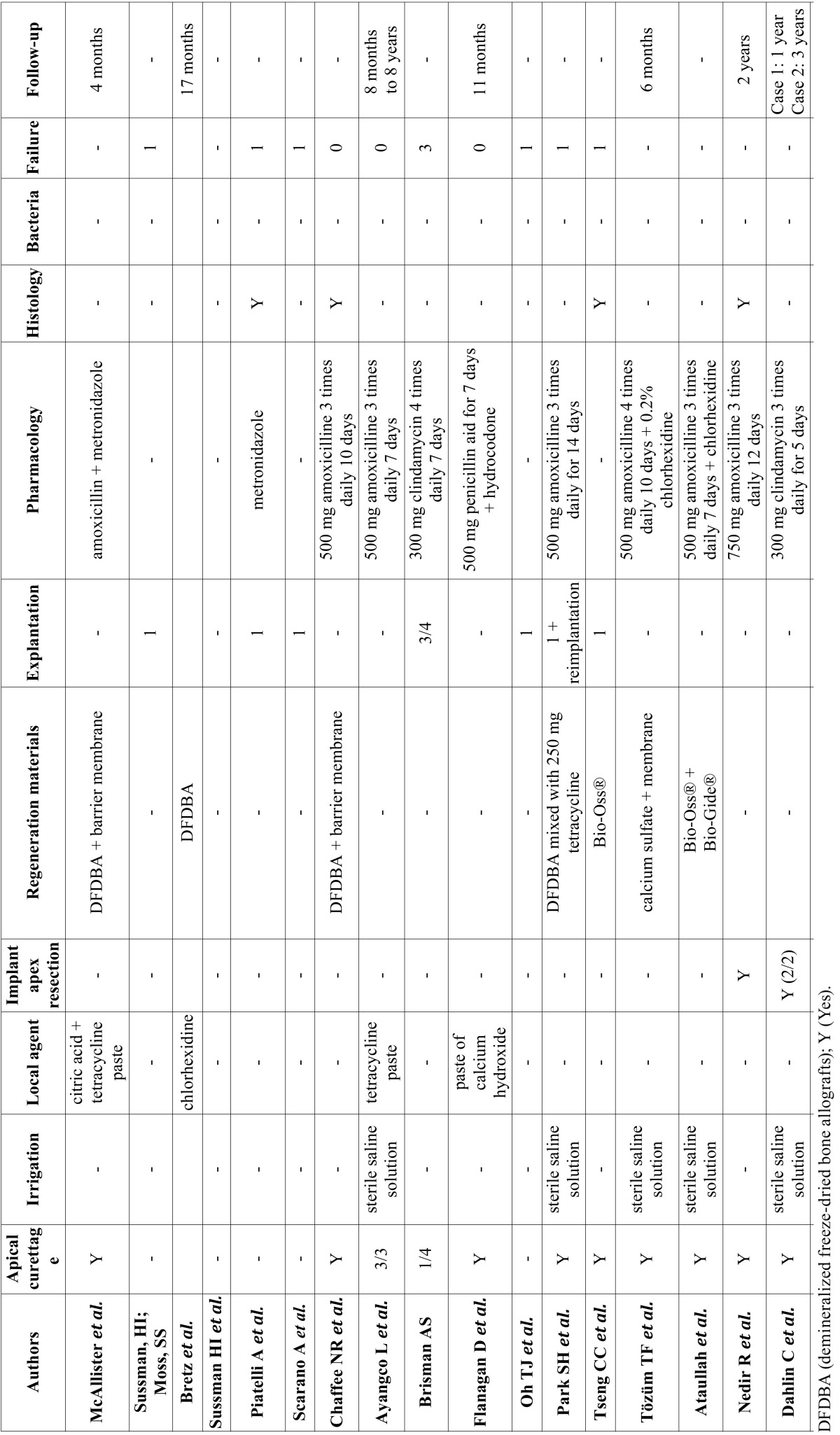
Articles with n<5, treatment and follow-up.

**Table 5 continue:**
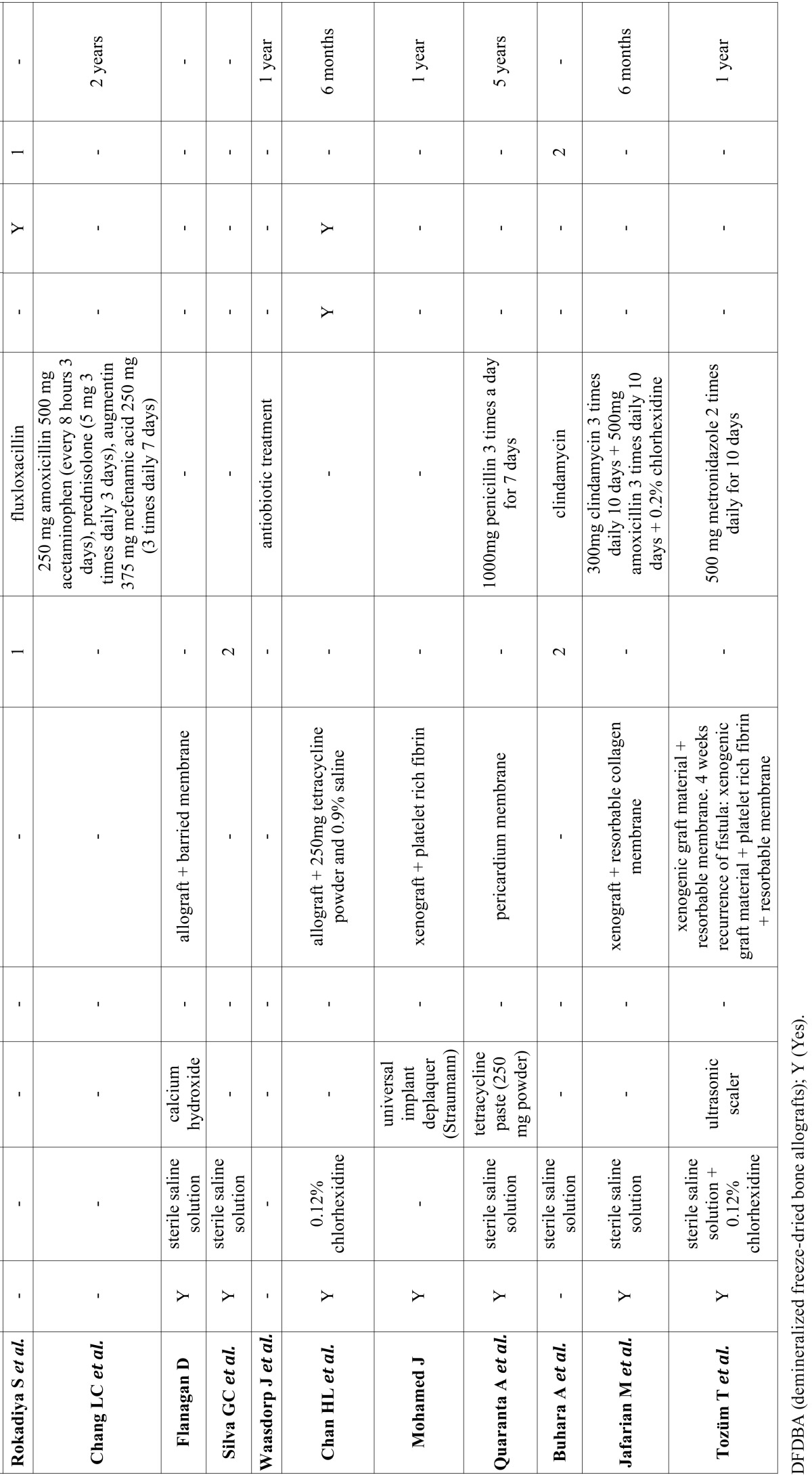
Articles with n<5, treatment and follow-up.

- Description of study characteristics 

 Table 2,  Table 3,  Table 4,  Table 4 continue,  Table 5,  Table 5 continue present details to study settings. Reviewers extracted the following variables from the selected manuscripts: authors, year, setting (university/hospital/privace practice), study design (retrospective/prospective), implants evaluated, periapical implant lesion, frequency, state prior tooth and the adjacent tooth, clinical symptoms (pain and puffiness), signs (swelling, fistula) and radiological findings, percussion sound, diagnosis, treatment applied, pharmacology, success, failure and follow-up.

- Risk of Biass and Quality Assessment

 Table 6 summarizes the quality of the studies, all articles (1,2,4,8,10-12,14-20) were classified as high bias.

**Table 6:**
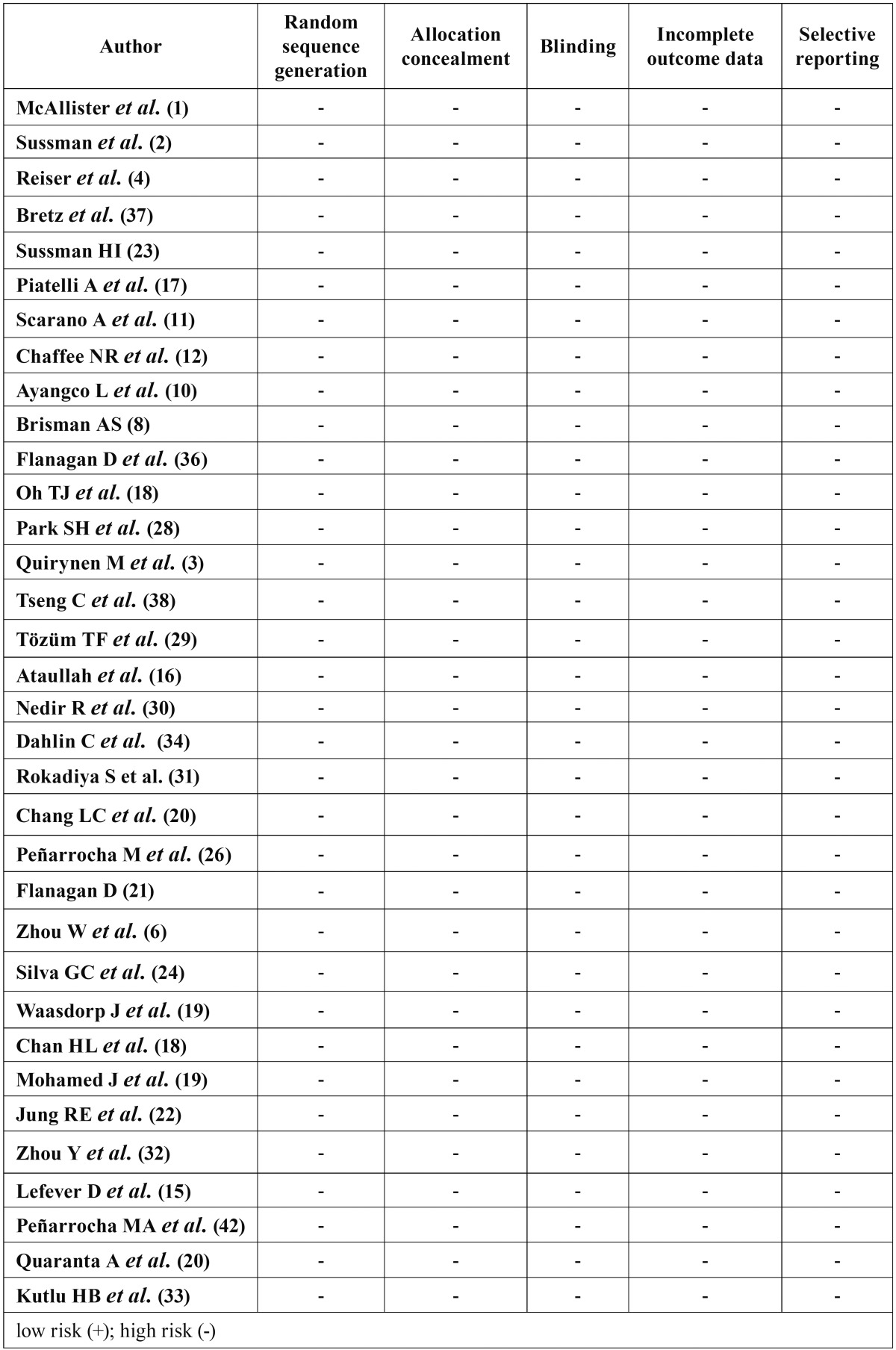
Assessment of the risk of bias.

- Frecuency

The frecuency of implant periapical lesion reported in the literature show considerable discrepancies between studies. Quirynen *et al.* (3), in a retrospective study on 539 implants, obtained an prevalence of 1.6% in maxilla and 2.7% in the mandible. Reiser & Nevins (4) found 10 cases in 3800 placed implants with an prevalence of 0.26%. Peñarrocha *et al.* (21) conducted a retrospective study including 5200 implants, of which 22 were diagnosed with implant periapical lesion, representing an prevalence of 0.4%. Zhou *et al.* (6) studied 128 implants with adjacent teeth that had received endodontic treatment, the incidence reported was 7.8%. Studies are required with more patients to provide more data on the frequency of periapical implant lesion.

- Etiology

Different etiological factors have been suggested for periapical implant lesion, although evidence is very limited. According to the source of contamination: a) contamination of the surgical bed: implant surface contamination (9,10), overhealing of bone during drilling (9,11). b) pre-existing pathology: immediate post-extraction placement (21), endodontic pathology associated with the extracted tooth or adjacent teeth (6,8,22), pre-existing bone pathology (6), and presence of root remains or foreign bodies (9,12).

Some authors (9,21) relate immediate implant placement after tooth extraction with apical pathology and the onset of periapical periimplantitis. Brisman *et al.* (8) associated the failure of four implants to the existence of adjacent endodontically treated teeth, which were asymptomatic and showed no radiographic signs of pathology. Lefever *et al.* (22) obtained statistically significance between the onset of apical peri-implantitis and the existence of neighboring teeth with signs of apical pathology compared to adjacent teeth without apical lesions. These authors suggest that the distance between teeth and implants is important for the development of these infections.

- Diagnosis

Diagnosis of implant periapical lesions involves clinical and radiographic assessments. The symptoms (pain and puffiness) and signs (swelling, fistula and drainage) may appear with different intensity depending on the stage of the lesion. Radiographically, radiolucency around the implant apex may be observed.

Regarding at the time that this condition is detected, the studies have described the radiological findings usually appear between 7 and 16 days after surgery (6,21,23), some cases described the appearance until 3 months after implant placement (11,16,24,25). Other authors (3,5,14,22,26-29) do not specify the exact timing of detection, they mentioned that the finding was before prosthetic loading.

- Clinical classification

No consensus exists about how to classify the lesion. Have been proposed four different classifications.

Reiser & Nevins (4) classified the injuries as inactive (not infected) and infected. Sussman (14) divided the nature of of lesions as follows: implant to tooth when produced during implant insertion (type I) and tooth to implant when implant apical lesion occurred due to infection of adjacent teeth to the implant that can be contaminated the apical part of the implant (type II). Kadkhodazadeh *et al.* (30) in 2013 introduced a new classification about relationship between periodontal, periapical, and peri-implant complications, only the condition of the implant apical lesion is applied, does not consider development time. Peñarrocha *et al.* (31) described the disease into 3 stages: non-suppurative; suppurative; and subacute. These stages are based on the similarity with the tooth periapical pathology.

In implants, the most important difference is that not exist periodontal ligament, implant fixation to bone differs. The non-suppurative phase has acute symptoms, spontaneous, moderate-severe, continuous, localized in apex implant, however, pain does not increase with the mastication. Mucosa can be swelled and reddish, implant percussion produces a tympanic sound, without increasing pain. No radiographic changes are observed in this initial phase.

In the suppurative phase, symptoms and signs are the same as in non-suppurated, but may appear periimplant radiolucent area radiographically.

Subacute phase is characterized by dull pain, with posible fistulous tract, buccal abscess or suppuration around the implant neck. Percussion produces a tympanic sound when the process fistulizes and the implant remains stable and a dull sound to percussion is produced when there is bone destruction around the implant, therefore, the implant may have mobility. Radiographically, the radiolucent area of the implant body may be associated with the destruction along the implant. The difference of this phase between the tooth and the implant, is that not having periodontal ligament, purulent material find the area with less pressure to drain along the axis of the implant, creating a detachment of the implant with a loss of fixation (31).

In a case series study (21) of 22 implant periapical lesion, described the most frequent stage of the disease as suppurative (60%) followed by the non-suppurative phase (35%) and the subacute phase (5%). The process is evolutional, from non-suppurative phase to subacute phase with losing fixation and implant failure.

- Treatment

● Pharmacology 

The following antibiotics have been used in the reviewed articles on treatment of implant periapical lesions: amoxicillin (5,10,12,23,27,32-36), amoxicillin/clavulanate (21), metronidazole (16,37,38) and clindamicime (8,39).

In some published case series (5,39), initial treatment with antibiotics was not effective to control symptomatic or active lesions, which required surgical access. Romanos *et al.* (40) concluded in their review that antibiotic treatment alone is not effective.

● Surgical procedure 

The surgical treatment comprises: infiltrative anesthesia, incision, full-thickness flap, osteotomy, apical curettage of granulation tissue and profuse irrigation.

Some authors after remove granulation tissue, irrigate with sterile saline solution (1,5,16) or chlorhexidine (6,24). Other agents have been suggested for topical decontamination of the implant surface, such as chlorhexidine (5,6,24), calcium hydroxide paste (17) or tetracycline paste (6,10,16,24), but there are no evidence of the efficacy of any of them.

Some studies reported the use of bone regeneration materials, accompanied or not with tissue regeneration barriers, in order to achieve complete bone regeneration of the defect (3,15,17,24,34,41). Sectioning of the implant apex has been suggested in those cases in which total removal of the granular tissue is not assured otherwise (16,24,39). Depending on the state of the adjacent pieces is recommended root-canal therapy or periapical surgery if the adjacent tooth was endodontically treated (6,34).

- Prognosis

The prognosis for these lesions is favorable, the literature describes a survival of 73.2% to 97.4% of the implants treated with a maximum follow-up of 4.5 years (5,21,22,42). Success depends on early diagnosis and adequate remaining bone fixation. The implementation of the new imaging technologies, CBCT, provide benefits in the early diagnosis, showing a clear clinical image of the periimplant bone loss (43).

## Discussion

The frequency of this lesion is low as described in the literature (3,4,6,21). Must be taken into account that the available articles are retrospective, another methodological design and the largest study of this lesion could increase its frequency within the early implant failures.

Regarding etiology, inflammatory-infectious origin can be delimited to factors of contamination of the surgical bed and/or pre-existing pathology. Oral surgery is a non-aseptic surgery, still using sterility protocols. Implant surface may become contaminated with saliva, epithelial cells or lubricant oil from the rotary material (9,10). Another factor that may be neglected during surgery is the overheating of the bone, due to an inadequate irrigation or an excessive time during the tilling of the surgical bed (9,11).

It is difficult to exclude the existence of remaining bone pathology, such as a residual cyst, after tooth extraction in the space where the implant will be placed. Neither periapical x-ray or panoramic radiograph are able to detect a radiolucent area if the injury does not destroy the osseous cortical (44). It is recommended the diagnosis with CBCT, however, it is not easy to diagnose small residual lesions in the bone.

The development of this lesion is early (6,21,23), so follow-up after implant placement is a key factor to properly diagnose and treat the pathological condition. Accordance with Peñarrocha *et al.* (45), therapeutic option is decided according to the evolution of the periapical implant lesion, based on clinical diagnosis and radiological explorations. Periimplant radiolucencies may be casual findings during routine radiographic assessments. If the patient is asymptomatic and the diameter of the radiolucent area is small, it is not necessary to treat the lesion; over-preparation of the implant bed is the most probable cause, and only periodic radiographic controls should be done. If in the controls, the radiolucency has increased in size or the patient develops pain, the surgical treatment will be applied.

If after placing an implant appears localized pain in periimplant area, with or without radiographic changes, should be considered as an inflammatory periapical implant pathology, acute non-suppurative or suppurative. In any of these cases, is indicated apical implant curettage to remove the granulation tissue.

Pharmacological treatment is based on antibiotics for at least one week, the combination of a broad spectrum antibiotic such as amoxycillin against anaerobes with another effective as metronidazole.

Limitations of the present study

Regard to the level of bias in the studies, several limitations should be considered about the design of the studies because it was not possible the application of quality questionnaires. First, all final articles obtained were a case report or case series, thus the risk of bias was high. Second, the low prevalence and limited knowledge of the lesion. Third, absence of homogeneity of data. It is necessary data collection protocol during the osseointegration phase for future studies.

## Conclusions

Evidence on the subject is very limited, currently etiology lacks consensus. If after placing an implant appears localized pain in periapical area, with or without radiographic changes, should be considered periapical implant pathology. The early diagnosis of periapical implant lesions during the osseointegration phase and early surgical treatment, will lead to a higher survival rate of implants treated, hence preventing the need for implant extraction.
